# Differences in Access to Services in Rural Emergency Departments of Quebec and Ontario

**DOI:** 10.1371/journal.pone.0123746

**Published:** 2015-04-15

**Authors:** Richard Fleet, Christina Pelletier, Jérémie Marcoux, Julie Maltais-Giguère, Patrick Archambault, Louis David Audette, Jeff Plant, François Bégin, Fatoumata Korika Tounkara, Julien Poitras

**Affiliations:** 1 Department of Family and Emergency Medicine, Laval University, Quebec, Quebec, Canada; 2 Research Chair in Emergency Medicine, Laval University—CHAU Hôtel-Dieu de Lévis Hospital, Lévis City, Quebec, Canada; 3 Faculty of medicine, University of British Columbia and Department of Emergency Medicine, Penticton regional Hospital, Penticton, British Columbia, Canada; 4 Department of Emergency Medicine, CSSS Alphonse Desjardins—Hôtel-Dieu de Lévis Hospital, Lévis, Quebec, Canada

## Abstract

**Introduction:**

Rural emergency departments (EDs) are important safety nets for the 20% of Canadians who live there. A serious problem in access to health care services in these regions has emerged. However, there are considerable geographic disparities in access to trauma center in Canada. The main objective of this project was to compare access to local 24/7 support services in rural EDs in Quebec and Ontario as well as distances to Levels 1 and 2 trauma centers.

**Materials and Methods:**

Rural EDs were identified through the Canadian Healthcare Association's Guide to Canadian Healthcare Facilities. We selected hospitals with 24/7 ED physician coverage and hospitalization beds that were located in rural communities. There were 26 rural EDs in Quebec and 62 in Ontario meeting these criteria. Data were collected from ministries of health, local health authorities, and ED statistics. Fisher’s exact test, the t-test or Wilcoxon-Mann-Whitney test, were performed to compare rural EDs of Quebec and Ontario.

**Results:**

All selected EDs of Quebec and Ontario agreed to participate in the study. The number of EDs visits was higher in Quebec than in Ontario (19 322 ± 6 275 vs 13 446 ± 8 056, *p* = 0.0013). There were no significant differences between Quebec and Ontario’s local population and small town population density. Quebec’s EDs have better access to advance imaging services such as CT scanner (77% vs 15%, *p* < .0001) and most the consultant support and ICU (92% vs 31%, *p* < .0001). Finally, more than 40% of rural EDs in Quebec and Ontario are more than 300 km away from Levels 1 and 2 trauma centers.

**Conclusions:**

Considering that Canada has a Universal health care system, the discrepancies between Quebec and Ontario in access to support services are intriguing. A nationwide study is justified to address this issue.

## Introduction

More than 6 000 000 Canadians live in rural areas [1] and a significant proportion of emergency department (ED) visits take place in rural hospitals [2–4]. Rural EDs are safety nets for rural citizens who are generally older, have greater health care needs and are at a proportionally higher risk of trauma and trauma death than their urban counterparts [5]. In the 1990s, most of Canada’s provinces undertook health care regionalization projects. The centralization of services in urban centers led to the closure of many small-town hospitals [6, 7].

As a result, health professionals and patients in rural areas must contend with reduced access to diagnostic tools and local specialized services [5, 8, 9]. This approach to constrain costs is debated [10] and, in certain cases, possible associations with adverse patient outcomes were uncovered [11, 12]. However, the actual level of services offered in rural EDs in Canada is unclear and despite a position paper on the subject by the CAEP in 1997 [13], there has been minimal research in rural emergency care issues. Our preliminary research suggests important interprovincial discrepancies in access to emergency support services that requires further investigation [14, 15].

The main objective of this project was to compare access to local 24/7 support services in rural EDs in the provinces of Québec and Ontario as well as distances to Level 1 and Level 2 trauma centers. This is the first study comparing access to emergency services in Canada’s largest and most populated provinces.

## Materials and Methods

The protocol was submitted to our internal ethics review board (CHAU Hôtel-Dieu de Lévis) for a nationwide pilot study. It was deemed not to require further ethics evaluation, based on the Tri-Council Policy Statement, as the research focused on the availability of services in public health-care facilities and did not involve human subjects.

### Selection of rural EDs

We focused on rural EDs with 24/7 physician coverage located in hospitals with access to hospital beds for acute admissions. To facilitate eventual comparisons with EDs elsewhere, we excluded community health centers or clinics, nursing stations, mobile health units, or private facilities. Decision on the definition of “rural” used in this study was derived from Statistics Canada’s definitions after consultation with professionals from its Division of Geography. We selected Statistics Canada’s “rural and small town” (RST) definition: “the population living in towns and municipalities outside the commuting zone of larger urban centers (i.e., outside the commuting zone of centers with a population of 10 000 or more)” [16]. The criteria for RSTs are presented in Table 1. EDs located in RST communities were then identified using the Guide to Canadian Healthcare Facilities [17]. The hospitals’ status was then confirmed with provincial health ministries by telephone or email. Furthermore, the list of RST where participating centers were located was also submitted to Statistics Canada for confirmation. There are 26 rural EDs in Quebec and 62 in Ontario.

**Table 1:** Criteria of the RST definition of Statistics Canada [16].

	Population	Population density
RST criteria 1	> 10 000	< 400 / km^2^
RST criteria 2	< 10 000	> 400 / km^2^
RST criteria 3	< 10 000	< 400 / km^2^

RST = rural and small town.

### Data collection

Data was collected between July 2010 and September 2013. They was gathered from various sources, including government databases, official websites (e.g., provincial health ministries, Ontario regional health authorities and Quebec “*Direction nationale des urgences”* and “*Direction des services préhospitaliers d’urgence”* [18]). Briefly, data on local access to 24/7 consultant support, imaging services and access to ICU beds were obtained by brief phone interviews (10 minutes) / questionnaires completed by ED managers. Data on ED patient volumes and number of stretchers were collected from official websites. Distance to designated Level 1 and Level 2 trauma centers (see Hameed *et al*. [19] for definition and identification of trauma centers) was calculated with Google Maps [20]. This Web-based mapping geographic information system provides valid estimations of road distances at low cost, has lower usability problems than other similar systems [21, 22], and has been used previously in health care research [22–25]. Finally, all sociodemographic variables were obtained from Statistics Canada website. Local population was defined as “population of the census subdivision” (i.e., Area that is a municipality or an area that is deemed to be equivalent to a municipality for statistical reporting purposes. Municipal status is defined by laws in effect in each province and territory in Canada) and number of population with school education or less was included “persons who had more that grade 10 educations and did not complete secondary school or receive a secondary school equivalency certificate or diploma” [26].

### Data entry and analysis

Data was entered and verified by two research assistants and analysed with SAS 9.4 (SAS Institute, Inc., Cary, North Carolina). Descriptive comparisons of socio-demographic and hospital characteristics, access to consultants and access to equipment using the Fisher exact test (proportions), the t-test (means) or Wilcoxon-Mann-Whitney test, were performed to compare rural EDs in Quebec and Ontario. Briefly, for all continuous variables with symmetric distribution such as median age of the population, median income, median income after tax, and annual ED patient visits, a parametric t-test was performed to compare the differences between emergency department groups. For all continuous variables with asymmetric distribution such as number of stretchers, local population, number of population with high school education or less and population density per square kilometer, a non-parametric Wilcoxon-Mann-Whitney test was performed to compare the differences between emergency department groups. Finally, for all categorical variables the Fisher’s exact test was performed to compare the differences between emergency department groups.

## Results

**Fig 1:**
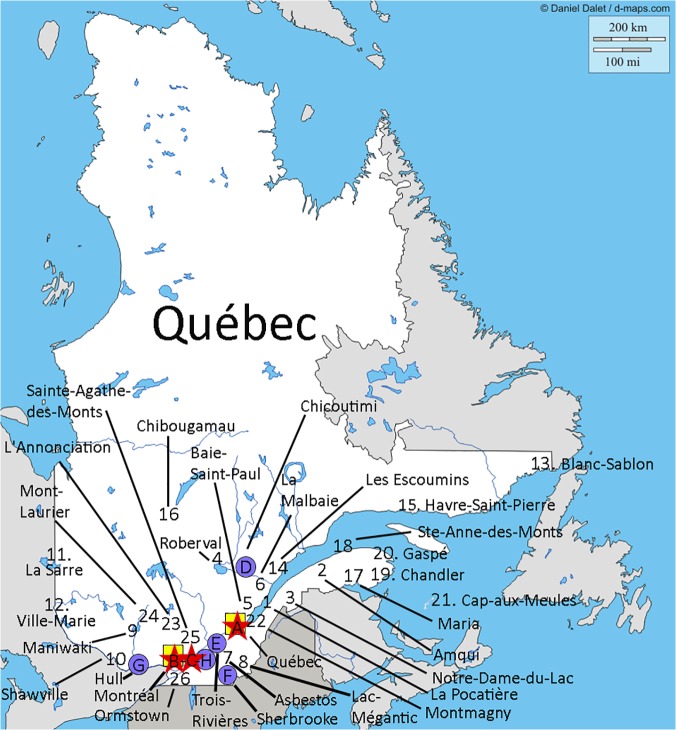
Location of RST EDs, Level 1 and 2 trauma centers, and airbases in Quebec. The Québec map was originally taken from http://d-maps.com/. All the maps are protected by copyright. They are free for any use, even commercial, in the following conditions: -The exact URL where the original map comes from must be mentioned -The number of used maps is limited to 10 for a publication (Web, DVD, book…) -The number of used maps is unlimited with BeGraphic GIS use. The cities where the 26 RST EDs are located are identified on the map. The red stars (A, B, C) represent the Level 1 trauma centers (refer to [18] for definition of trauma centers). The blue circles (D, E, F, G, H) represent the Level 2 trauma centers (refer to [18] for definition of trauma centers). The yellow square (A, B) represent the location of the airbases. Trauma centers: A = Hôpital de l'Enfant-Jésus (CHU de Québec); B = Hôpital du Sacré-Cœur de Montréal; C = Montreal General Hospital; D = Hôpital de Chicoutimi; E = Centre Hospitalier Régional de Trois Rivière; F = Hôpital Fleurimont (CHU de Sherbrooke); G = Hôpital de Hull; H = Hôpital Charles-Lemoyne.

**Fig 2:**
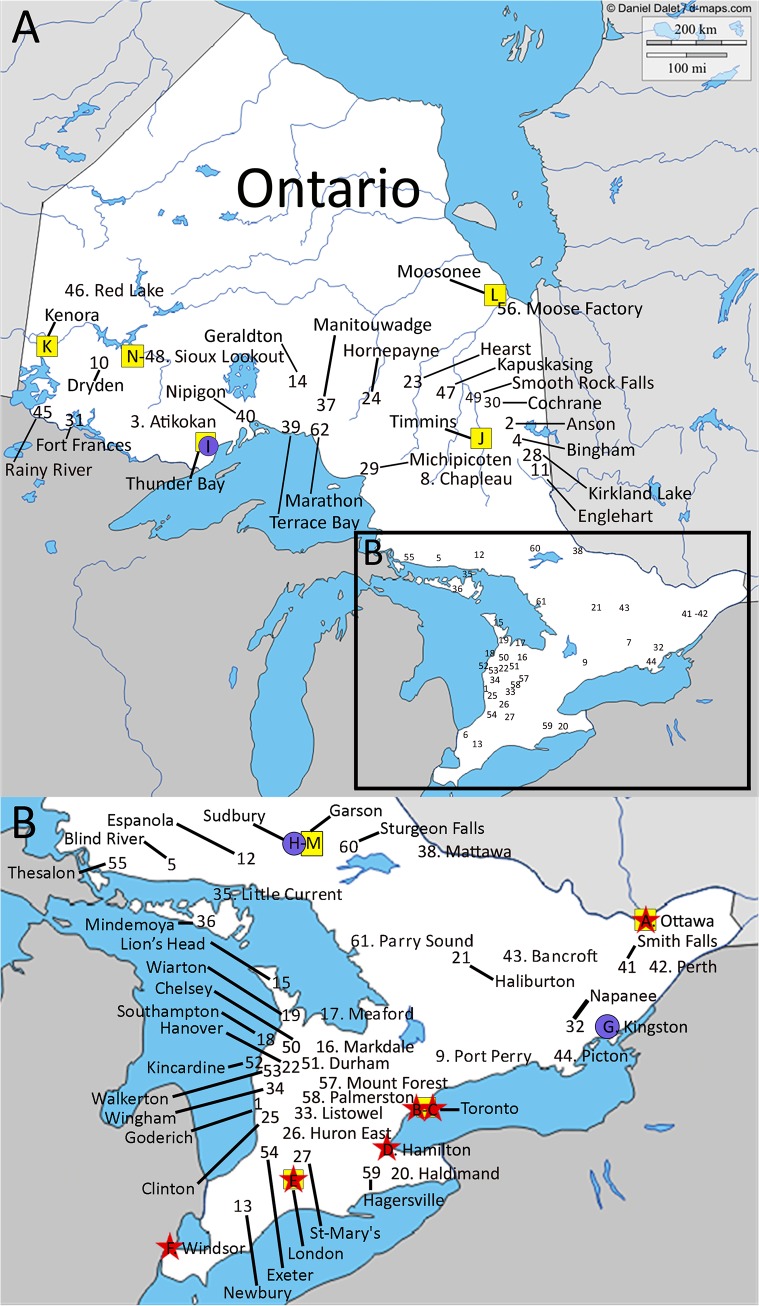
Location of RST EDs, Level 1 and 2 trauma centers, and airbases in ON. The Ontario map was originally taken from http://d-maps.com/. All the maps are protected by copyright. They are free for any use, even commercial, in the following conditions: -The exact URL where the original map comes from must be mentioned -The number of used maps is limited to 10 for a publication (Web, DVD, book…) -The number of used maps is unlimited with BeGraphic GIS use. The cities where the 62 RST EDs are located are identified on the map. The red stars (A, B, C, D, E, F) represent the Level 1 trauma centers (refer to [18] for definition of trauma centers). The blue circles (G, H, I) represent the Level 2 trauma centers (refer to [18] for definition of trauma centers). The yellow square (I, J, K, L, M, N) represent the location of the airbases. Trauma centers: A = The Ottawa Hospital—Civic Campus; B = St. Michael's Hospital; C = Sunnybrook Health Sciences Centre—Bayview Campus; D = Hamilton Health Sciences—Hamilton General Hospital; E = London Health Sciences Centre—Victoria Hospital; F = Hotel-Dieu Grace Hospital; G = Kingston General Hospital; H = Sudbury Regional Hospital—St. Joseph's Health Centre; I = Thunder Bay Regional Health Sciences Centre.

The study had a 100% participation rate. Twenty-six EDs were identified in Quebec and 62 in Ontario that met our criteria Figs 1 and 2 displays the location of RST EDs, Level 1 and 2 trauma centers, and airbases for each province.

Sociodemographic characteristics of the RST in which the EDs were located are presented in Table 2. There were no significant differences between Quebec and Ontario’s rural small town local populations and population density (for the studied EDs). Ontario’s rural local population had higher income and education than Quebec (*p* <.0001).

**Table 2:** Sociodemographic characteristics of the rural small towns in which the EDs were located.

	Quebec	Ontario	
Sociodemographic Characteristic	Mean ± SD	Mean ± SD	*p*-Value
Local population	6 442 ± 5 090	10 131± 13 259	0.2532^*^
Population density per square kilometer	117 ± 181	119 ± 234	0.2880
Median age of the population	45 ± 3	44 ± 4	0.1846^**^
Number of population with school education or less	1773 ± 1491	8248 ± 10705	<.0001
Median income ($)^¶^	22 410 ± 2 887	26 033 ± 4 056	<.0001
Median income after tax ($)^¶^	21 024 ± 2 269	23 582 ± 3 017	0.0002

Data from Statistics Canada [26].

^*^ *p*-Value from Wilcoxon-Mann-Whitney test;

^**^ *p*-Value from t-test;

^¶^ People 15 years of age and over; SD = Standard Deviation.

The general characteristics of the rural EDs and the results concerning 24/7 access to services and consultants are presented in Tables 3–5. To summarize, annual ED patient volumes and the number of ED stretchers are greater in Quebec than those of Ontario (p <0.001). The vast majority of rural EDs have local access to basic laboratory and X-ray services on a 24/7 basis. However, there were important differences in terms of access to advanced imaging services such as CT scanner (77% vs 15%, *p* <. 0001), consultant support such as Surgeon (81% vs 34% *p* <. 0001), pediatrician (12% vs 3% *p* < 0.1508), obstetrician-gynecologist (38% vs 6% *p* = 0.0005), and ICU care (92% vs 31% *p* <. 0001) with Quebec providing more comprehensive access in this respect. Forty-two percent of RST EDs in Quebec and 47% in Ontario are more than 300 km away from a Level 1 trauma center and 46% of RST EDs in Quebec and 63% in Ontario are more than 300 km from a Level 2 trauma center.

**Table 3:** Comparison of rural hospitals general characteristics.

	Quebec (*n* = 26)	Ontario (*n* = 62)	*p*-Value
Annual ED patient visits^1^	19 322 ± 6 275	13 446 ± 8 056	0.0013^*^
Number of stretchers^2^	11 ± 5	7 ± 3	<.0001^**^
Local ICU	92% (*n* = 24/26)	31% (*n* = 19/62)	<.0001^***^
EDs > 300 km of the nearest Level 1 trauma center	42% (*n* = 11/26)	47% (*n* = 29/62)	0.8156^***^
EDs > 300 km of the nearest Level 2 trauma center	46% (*n* = 12/26)	63% (*n* = 39/62)	0.1631^***^

^1^Average annual ED patient visits and standard deviation;

^2^ Average number of stretchers and standard deviation. ICU = intensive care unit; ED = emergency department.

^*^ *p*-Value from t-test;

^**^ *p*-Value from Wilcoxon-Mann-Whitney test;

^***^ *p*-Value from fisher exact test.

**Table 4:** Comparison of local 24/7 access to consultants in rural EDs.

	Quebec (*n* = 26)	Ontario (*n* = 62)	*p*-Value^*^
Psychiatrist	48% (*n* = 11/23)^$^	3% (*n* = 2/62)	<.0001
Anesthesiologist	69% (*n* = 18/26)	36% (*n* = 16/44)^&^	0.0128
Surgeon	81% (*n* = 21/26)	34% (*n* = 21/62)	<.0001
Internist	42% (*n* = 11/26)	11% (*n* = 7/62)	0.0026
Obstetrician-gynecologist	38% (*n* = 10/26)	6% (*n* = 4/62)	0.0005
Orthopedics	15% (*n* = 4/26)	10% (*n* = 6/62)	0.4733
Pediatrician	12% (*n* = 3/26)	3% (*n* = 2/62)	0.1508
Neurologist	0% (*n* = 0/23)^$^	2% (*n* = 1/62)	1.0000

^$^ The proportions were calculated for 23 EDs because of 3 missing value.

^&^ The proportions were calculated for 44 EDs because of 18 missing value.

^*^ *p*-Value from Fisher exact test.

**Table 5:** This is the Table 5. Comparison of 24/7 local access to equipment in rural EDs.

	Quebec (n = 26)	Ontario (n = 62)	*p*-Value^*^
Ultrasound	30% (n = 7/23)^**^	46% (n = 28/61)^***^	0.2249
Bedside ultrasound	81% (n = 21/26)	63% (n = 39/62)	0.1340
CT scanner	77% (n = 20/26)	15% (n = 9/62)	<.0001
Laboratory	100% (n = 26/26)	94% (n = 58/62)	0.3148
Magnetic resonance imaging	0% (n = 0/26)	0% (n = 0/61)	
Basic X-rays	92% (n = 24/26)	92% (n = 57/62)	1.000

^**^ The proportions were calculated for 23 EDs because of 3 missing value.

^***^ The proportions were calculated for 61 EDs because of 1 missing value.

^*^ *p*-Value from Fisher exact test.

## Discussion

Results show that Ontario has almost three times more rural EDs providing 24/7 physician coverage with acute care hospitalization beds. However, Ontario’s rural EDs have proportionally fewer CT scanners, and are supported by limited consultant services and in hospital ICUs as compared to Quebec.

A comparative study of emergency support services in Quebec and Ontario is of interest as both provinces share similar geo-demographic attributes and health care spending patterns. Quebec is the largest province in terms of area (1,542,056 km^2^) [27], and is the second most populated (population: 8,054,800) [28]. A total of 19% of Quebec’s population lives in rural areas [1]. Ontario (population 13,505,900 [28]) is the second largest province in terms of land area (1,076,395 km^2^) [27], and 14% of the population lives rurally [1]. Both provinces report comparable per capita annual health care cost: Quebec = $5,469 and Ontario = $5,849 [29]. Finally, these provinces ranked almost equally on the 2013 Provincial Healthcare Index report [30]. Using data from the Canadian Institute for Health Information (CIHI), this index measures the provision of healthcare in each province and is captured using 46 indicators, aggregated into four broad components: 1) availability of resources; 2) use of resources; 3) access to resources and 4) clinical performance of medical goods and services [30]. When compared to other provinces, Quebec received the best value for money from its public healthcare system, followed by Ontario [30]. For these reasons, a comparison between these neighboring provinces is of relevance.

So, how can we explain the differences between Quebec and Ontario? First, the higher number of rural EDs in Ontario may, in part, be a reflection of Ontario’s larger population. It is possible that the operational costs and staffing challenges associated with the larger number of facilities may be barriers to providing more widespread access to CT scanning, and specialized care. Second, Ontario and Quebec have different EMS systems (training, scope of practice, management, private versus public, and air medevac capacity, etc.) that may facilitate inter-facility transfers in of Ontario. This study did not address this issue in detail however. Third, for critical patients, Ontario has the Criticall system, which is able to draw upon of the entire network of services to help physicians access the support they need for their patients. The Criticall service is available to physicians who are caring for critically ill adults and children, as well as neonates. From 2008 to 2009, Criticall managed approximately 15,319 cases and 5,771 [31]. Thus, a well-integrated critical care system of Ontario with advanced EMS may also offer an explanation for the different levels of rural resources, particularly ICUs. Finally, different provincial policy may also explain some of the discrepancies. Quebec is, to our knowledge, the only province to have an ED management guide [32]. This guide, reflecting government policy, makes recommendations regarding the services that should be provided by Quebec’s urban and rural emergency departments. For example, it recommends that EDs with more than 10,000 visits per year (requiring stretchers) have a local CT scanner, surgical critical care capability [32]. In theory, Quebec’s policy main explain the significant levels of rural resources offered. We are currently conducting a study to examine the actual implementation of the guide’s recommendations in QC’s rural EDs [33].

The provision of 24/7 emergency care in rural areas is a challenge. The question of what services are required to provide quality time-sensitive emergency care has not been determined. Yet, the observed differences reported here in terms of access, in particular to a CT scanner, general surgery is at the very least, intriguing. Especially, in the context of Canada’s Universal health system and the Canadian Health Act’s (CHA) requirement of ‘‘reasonable access” to health services [11].

Access to a CT scanner, now a ubiquitous tool in urban hospitals in Canada and in nearly all US rural EDs [34] may improve timely diagnoses, treatments and appropriateness of referrals and transfers in particular for life-threatening conditions such as stroke, head trauma and other surgical emergencies. General surgeons, also present in 87% of US Critical Access Hospitals (mostly small and rural hospitals) [35], have a vast array of skills and may provide life-saving care in trauma cases, thus potentially improving on the recently reported concerning mortality rates for trauma in rural settings. A local surgical service may also minimize inter-facility transfers for common surgical emergencies, assist emergency physicians in procedures and provide backup surgical care for other surgical specialties such as obstetrics [36]. Ultimately, a nationwide study of the relationship between levels of resources/costs, interfacilty transfer requirements on patient outcomes is required to help clarify what model of rural emergency care would be is better.

While awaiting Canadian nationwide studies with outcome measures, decision makers can take note of US data that suggest poor outcomes for rural citizens. Specifically, recent reports showed that rural hospitals (from the critical access hospital program) had fewer clinical capabilities, worse processes of care, and higher mortality rates for acute myocardial infarction, congestive heart failure and pneumonia and ischemic stroke [35, 37, 38]. Similar studies are required in Canada especially in the light of these findings of the limited resources offered in Canadian rural hospitals.

Despite the challenges in providing access to emergency care as well as the different approaches offered, both Quebec and Ontario have displayed significant leadership in rural emergency care. Ontario was the first province in Canada to invest in a CT scan in a rural community and publish on its favorable impact [9]. Researchers from Ontario have also pioneered the first studies describing the need and reasons for inter-facility transfers in relation to local access to service [39]. Ontario also has developed Criticall to improve the transfer of critical care patients, Quebec has displayed leadership with its Emergency department management Guide and associated policy that was developed with the goal of making all stakeholders accountable for quality of care in EDs and more or less assure that rural EDs are well equipped and staff.

### Limitation

This study has certain limitations. To minimize ED managers’ time collecting data, we focused on major services and ED characteristics. We did not collect data on access to other services in the community such as family doctors, medical clinics, and long-term care facilities. These services obviously affect ED consultation volume. For the purpose of this study, we did not examine inter-facility transfer data and patient outcomes.

This study relied on information provided through questionnaires and brief telephone interviews with local health-care providers. Information was obtained mainly from one source, and cross-checking with other databases was not always possible. However, the information requested was straightforward and easily accessed by professionals working in these small RST EDs. Furthermore, to the best of our knowledge, the information obtained on 24/7 access to services in this study is not easily or reliably accessible through standardized databases in Canada [40].

Another limitation of our study is the lack of travel time estimates to Level 1 and 2 trauma centers in combination with the distance estimations. Total inter-facility transfer times (call from rural EDs to arrival at Level 1 and 2 Trauma center) would better represent the care pathways of these patients factoring in weather, geography, roadwork, and of course overall transport capabilities. We are planning such a study.

Finally, we reported population statistics on the RSTs where the hospitals were located. We did not obtain data on the hospitals’ service areas. It would have been interesting to estimate the populations and the size of the territories served by theses rural hospitals. Experience with this information is that it is difficult to reliably obtain and verify [15, 41–43].

## Conclusions

Our results suggest that major differences exist in access to support services in rural EDs between Quebec and Ontario. From a benchmarking perspective, this study should help decision-makers and health care professionals reflect on current resource allocations for the provision of safe and equitable rural emergency care. A nationwide study is required to further understand the observed geographic discrepancies and their impact of patient outcomes that are intriguing in the context of Canada’s universal health care program.
